# 

**Published:** 1892-09

**Authors:** 


### CONTENTS.

ORIGINAL COMMUNICATIONS—

The Management of Post-Partal Hemorrhage. By Prof. Grynfelt, France........ 385
The Artificial Feeding of Infants. By Charles S. Shaw, M. D., Pittsburg, Pa.... 389
Electricity as a Therapeutical Means in the Treatment of Lateral Spinal Curvature. By Hugh Hagan, M. D., Atlanta, Ga............................ 394
A Case of Cerebro Spinal Hyperæmia. By A. C. Moreland, M. D., Atlanta, Ga ... 397

SELECTED ARTICLE—

The Present Status of the Surgery of the Vermiform Appendix. By John A. Wyeth, M. D., New York.............................................. 400

SOCIETY REPORT—

Allegheny County Medical Society .............................................. 408

CORRESPONDENCE—

The Treatment of Genito-Urinary Diseases in New York City................. 413

EDITORIAL—

Something about the Journal.................................................. 417
The End of the Mitchell-Ward Case.......................................... 420
The Cholera.......................................................................... 422
The Money-Value Again.......................................................... 424

SELECTIONS.......................................................................... 425
MEDICAL ITEMS.................................................................... 442
BOOK REVIEWS...................................................................... 445
PAMPHLETS RECEIVED............................................................ 448
INDEX TO ADVERTISEMENTS.................................................... xii
ITEMS OF INTEREST............................................................... xi

